# Optimal aspiration pressure of suction pump for oocyte retrieval in infertile patients undergoing in vitro fertilization

**DOI:** 10.1371/journal.pone.0317812

**Published:** 2025-01-27

**Authors:** Sung Woo Kim, Hye Yun Kim, Ji Yeon Han, Hoon Kim, Seung-Yup Ku

**Affiliations:** 1 Department of Obstetrics and Gynecology, Seoul National University Hospital, Seoul, South Korea; 2 Department of Obstetrics and Gynecology, Seoul National University College of Medicine, Seoul, South Korea; 3 Institute of Reproductive Medicine and Population, Medical Research Center, Seoul National University, Seoul, South Korea; Western Michigan University School of Medicine: Western Michigan University Homer Stryker MD School of Medicine, UNITED STATES OF AMERICA

## Abstract

**Background:**

The oocyte retrieval is a critical step in assisted reproductive technologies, including in vitro fertilization and fertility preservation. Despite evolving techniques, the optimal aspiration pressure during retrieval remains debatable, with limited in vivo human studies. Existing studies, primarily in vitro and on animals, suggest that inappropriate aspiration pressures can impair oocyte quality. This study aims to compares the effects of two different aspiration pressures, 120mmHg and 150mmHg, on oocyte recovery, damage, and subsequent embryo development and pregnancy outcomes in infertile women undergoing transvaginal ultrasound-guided oocyte retrieval.

**Methods and findings:**

This retrospective study analyzed data from 891 women at Seoul National University Hospital between May 2018 and August 2023. A total of 400 cycles were included, with 202 at 120 mmHg and 198 at 150 mmHg aspiration pressures. The primary outcomes were the number of retrieved, matured, fractured oocytes, embryos, and good-grade embryos. Pregnancy outcomes were evaluated by comparing the clinical pregnancy rates and live birth rates. Univariate and multivariate logistic regression analyses were conducted to evaluate the relationship between aspiration pressure, clinical pregnancy, and live birth rates. There was statistically significant difference in the number of retrieved oocytes and mature oocytes between the 120 mmHg group and the 150 mmHg group (6.3±5.2 vs. 7.7±6.7, p = 0.018; 4.4±3.7 vs. 5.6±5.3, p = 0.011). The number of embryos and good grade embryos also differed significantly (3.3±3.1 vs. 4.2±3.9, p = 0.011; 1.0±1.6 vs. 1.5±2.6, p = 0.031). However, there were no significant differences in clinical pregnancy and live birth rates between the two groups in multivariate logistic regression analysis (adjusted OR = 0.725, p = 0.519; adjusted OR = 0.370, p = 0.170).

**Conclusions:**

Increasing the aspiration pressure to 150mmHg led to a higher yield of oocytes and embryos than 120mmHg, without any negative impact on oocyte quality or live birth rates. These findings provide valuable insights for clinical decision-making in infertility treatments, suggesting that 150mmHg may be a more effective pressure for oocyte retrieval in in vitro fertilization and embryo transfer.

## Introduction

The oocyte retrieval is an essential procedure to perform in vitro fertilization (IVF) for infertility treatment or to preserve fertility through oocyte cryopreservation or embryo cryopreservation [1]. When collecting oocytes, there are two methods for applying negative pressure to the aspiration needle: manual aspiration and pump aspiration [1, 2]. In the early 1970s, manual aspiration was performed by connecting a plastic extension tube and plastic syringe to the aspiration needle [2]. In the mid-1970s, a special aspiration unit consisting of an aspiration needle, tubing collection tube, tubing, thumb valve, and vacuum bottle with adjustable pressure was designed, making it possible to more precisely control the aspiration process of follicles by turning the thumb valve on and off manually [3]. In the 1980s, a foot-operated vacuum pump set to a pressure of 100 mm Hg further improved the oocyte recovery rate [4]. Since then, people have primarily used digital pumps, which enable aspiration to the set pressure by adjusting the pressure through the digital number pad and then pressing the foot pedal [5].

Manual aspiration provides a cost advantage because it requires no additional equipment beyond a syringe. However, it has drawbacks: the pressure may be excessive depending on the force applied to the syringe and its volume, and the pressure is inconsistent throughout the aspiration process [6, 7]. If the aspiration pressure is excessive, it increases the shear force of the laminar flow. Conversely, if the aspiration pressure is not constant, it leads to turbulent flow within the aspiration system, potentially increasing the risk of oocyte damage due to the removal of its cumulus mass or fracture of the zona [8]. On the other hand, digital pump aspiration has the advantage of being able to perform aspiration at a constant pressure and monitoring the pressure in real time. However, if the aspiration pressure is too low during pump aspiration, it fails to separate the cumulus oophorus complex (COC) from the follicle, thereby reducing the oocyte recovery rate. Conversely, if the aspiration pressure is too high, it can cause physical damage to the separated COC, so optimal pressure is important [9].

Despite the importance of the optimal aspiration pressure of the suction pump during oocyte retrieval, the majority of studies have focused on animals in vitro, with a dearth of in vivo human studies [6, 8, 10, 11]. The number and quality of retrieved oocytes significantly influence embryo quality post-fertilization and the likelihood of successful pregnancy after embryo transfer [12–14]. As the aspiration pressure of the suction pump can affect oocyte recovery, oocyte quality, and resultant embryo quality, determining the optimal pressure is crucial for improving IVF-ET outcomes.

There is no established consensus on proper aspiration pressure, despite the common use of off-the-shelf follicle aspiration devices. The 2019 ESHRE guideline notes that optimal aspiration pressure levels remain inconclusive, with pressures ranging from 100 mmHg to 200 mmHg in practice [1]. According to some research, the aspiration pressure should stay below 120 mmHg. This is because all the oocytes in studies using bovine ovaries lost their cumulus mass when they were suctioned at 150 mmHg with a 17-gauge needle (1.0 mm inner diameter) [8, 15]. However, these findings may not directly translate to human in vivo oocyte aspiration.

Therefore, this study aims to compare oocyte recovery, oocyte damage, embryo development, and pregnancy outcomes, including live birth rates, between 120mmHg and 150mmHg aspiration pressure groups during transvaginal ultrasound-guided oocyte retrieval in infertile women.

## Materials and methods

### Study subjects

**Fig 1:**
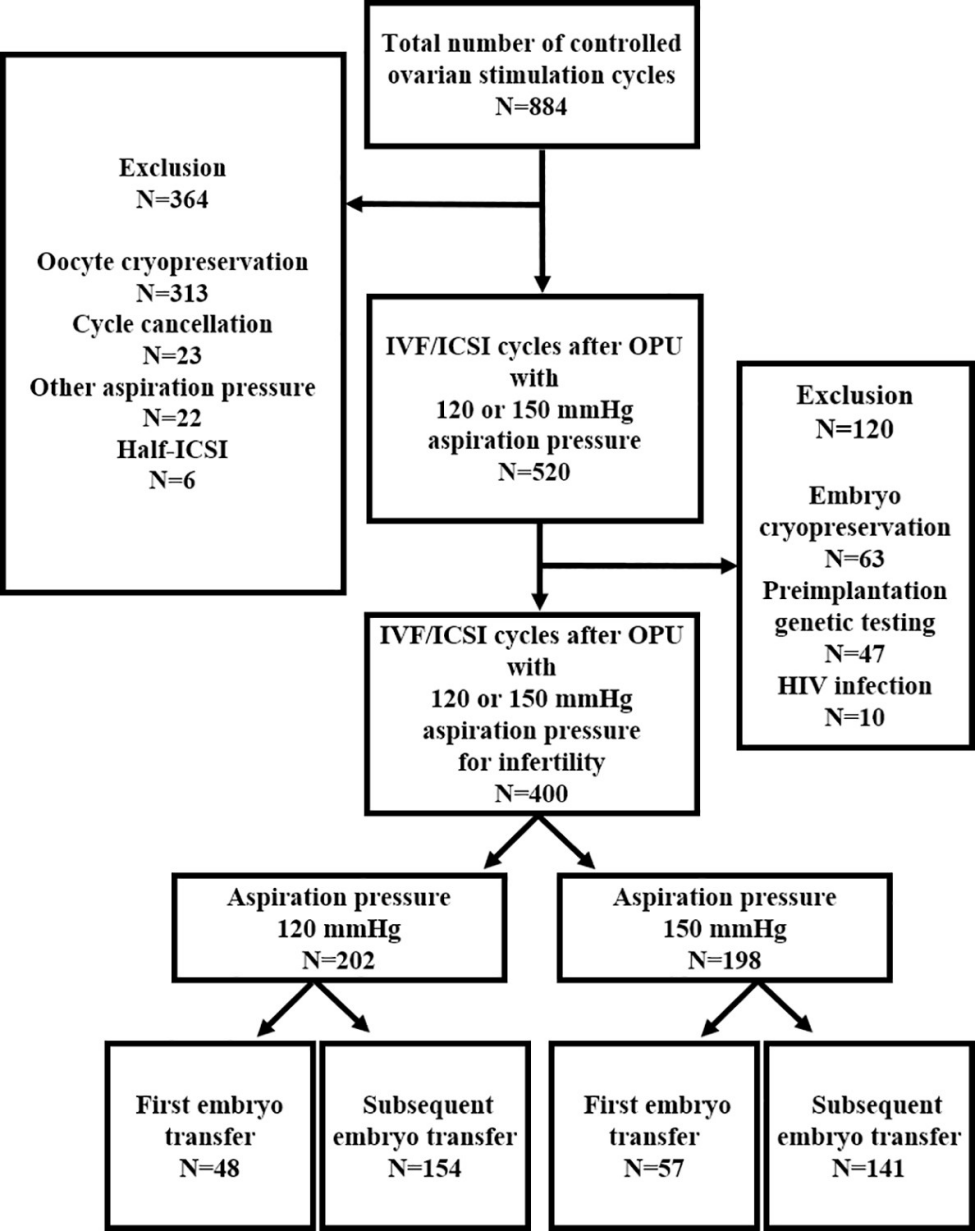
Patient inclusion and group assignment for aspiration pressure comparison.

This retrospective study was conducted at Seoul National University Hospital using medical records from 891 women who underwent ovarian stimulation between May 1, 2018 and August 31, 2023. The total number of Controlled ovarian stimulation (COS) cycles was 884 during the study period. The exclusion criteria are as follows: oocyte cryopreservation, cycle cancellation, use of aspiration pressures other than 120 or 150 mmHg, half-intracytoplasmic sperm injection (ICSI) cycles, embryo cryopreservation for fertility preservation, preimplantation genetic testing, and human immunodeficiency virus (HIV) infection. Finally, 400 cycles of IVF or ICSI with either 120 or 150 mmHg aspiration pressure to treat infertility were included in the analysis. The data were divided into two groups based on aspiration pressures of 120 mmHg and 150 mmHg. The aspiration pressure was determined based on the preference of the infertility specialist performing the procedure, set at either 120 mmHg or 150 mmHg. At our center, the infertility specialist who performs the oocyte retrieval is determined depending on the day of the week when the infertile patient receives outpatient treatment. For embryo transfer outcomes, each pressure group was further subdivided into two subgroups: those undergoing their first embryo transfer cycle and those undergoing subsequent embryo transfer cycles (Fig 1).

### Assisted reproductive treatment protocol

Patients undergoing assisted reproductive treatment (ART) cycles were administered recombinant follicle-stimulating hormone (FSH) alone (Gonal-F, Merck Serono, Switzerland). For poor responders, recombinant Luteinizing hormone (LH) (Luveris, Merck Serono, Switzerland) was added to the FSH treatment. This protocol was based on individual patient needs and clinician assessment. Ovarian stimulation was performed using either a GnRH antagonist protocol or a GnRH agonist long protocol. In the GnRH antagonist protocol, ovarian follicles were monitored using transvaginal ultrasonography, and when the leading follicle reached a size of 14mm, a GnRH antagonist (Cetrotide, Merck Serono, Switzerland) was initiated at a dose of 0.25mg daily to prevent premature ovulation. For the GnRH agonist long protocol, the GnRH agonist (Decapeptyl, Ferring Pharmaceuticals, Switzerland) was started at a dose of 0.1mg daily in the mid-luteal phase of the previous menstrual cycle.

When at least two follicles reached a diameter of 18mm, final oocyte maturation was triggered with 250μg of recombinant human chorionic gonadotropin (hCG) (Ovidrel, Merck Serono, Switzerland). Oocyte retrieval was performed 36 hours later under transvaginal ultrasound guidance using a 17-gauge needle (Cook Medical, USA) connected to a portable suction pump (Cook Medical, USA) set at either 120 mmHg or 150 mmHg. The collected oocytes were then fertilized either by conventional IVF or ICSI, depending on the individual case.

### Embryo transfer

The embryos were cultured for 3 or 5 days and graded to describe their quality as “good”, “moderate”, or “poor” according to modified Veeck’s classification which is used for the cleavage-stage embryos and blastocysts [16, 17]. For fresh transfers, luteal support with Crinone gel (Merck Serono, Switzerland) started on oocyte retrieval day: once daily for women ≤35 years, twice daily for >35 years. For frozen-thawed transfers, natural or hormone replacement therapy (HRT) cycles were used. In natural cycles, monitoring began on day 3 of menstruation. Transvaginal ultrasonography was performed every 2–3 days to track follicular development and endometrial thickness and monitored serum estradiol, progesterone, and LH levels during these visits. LH surge was defined as a 180% increase from the previous day. Embryo transfer occurred 5 days post-surge for Day 3 embryos, 7 days for blastocysts. Luteal support with once-daily Crinone started on transfer day. In HRT cycles, oral estradiol valerate (Progynova, Bayer, Germany) started at 4 mg daily, increasing to 6 mg after 4 days, then 8 mg after another 4 days. Crinone (twice daily) began when endometrial thickness reached 8 mm. Embryo transfer was performed on day 5 of progesterone for cleaved embryos and day 6 for blastocysts. Luteal support continued until beta-human chorionic gonadotropin(β-hCG) test in all cycles. HRT cycles also maintained estradiol valerate (8 mg daily).

### Outcomes

The number of retrieved, matured, and fractured oocytes were compared between the 120 mmHg and 150 mmHg pressure groups. Oocytes were classified as fractured based on microscopic examination of the zona pellucida; oocytes with visible breaks or damages to the zona pellucida were considered fractured. Additionally, the number of embryos and good-grade embryos were compared. For the results of embryo transfer cycles and pregnancy outcomes, only the first embryo transfer cycles were compared between the 120 mmHg and 150 mmHg pressure groups. The clinical pregnancy rate was defined as detecting a gestational sac on ultrasound, while the live birth rate was defined as the number of deliveries resulting in a live birth.

### Statistical analysis

The sample size calculation was based on the primary outcome, the number of retrieved oocytes. The effect size of 0.679 was derived from a previous study by Kumaran et al., calculated using Cohen’s d based on the mean number of oocytes retrieved and standard deviations reported in their 120 mmHg and 140 mmHg groups [18]. Using G*Power 3.1.9.7 (Heinrich-Heine-Universität Düsseldorf, Germany), with this effect size, a power of 95%, and a two-sided alpha of 0.05, the required total sample size was determined to be 116 (58 per group). The normality of data distribution was assessed. Continuous variables were analyzed using a Student’s t-test and presented as means ± standard deviations. The Chi-square test was used for categorical variables analysis, with Fisher’s exact test applied when the expected cell frequency was less than 5. Univariate and multivariate logistic regression analyses were conducted to evaluate the relationship between aspiration pressure, clinical pregnancy, and live birth rates while controlling for relevant confounding factors. Univariate regression models are presented as odds ratios (ORs) with 95% confidence intervals (CIs), and multivariate regression models are presented as adjusted odds ratios (aORs) with 95% confidence intervals (CIs). Statistical analysis was performed using IBM SPSS 27. Statistical significance was defined as p <0.05.

### Ethics statement

This study was approved by the Institutional Review Board (IRB) of Seoul National University Hospital (Approval No. 2210-015-1366) and was conducted by reviewing and analyzing de-identified patient records. The IRB granted a waiver for the requirement of informed consent based on the study’s reliance on anonymized data, ensuring that individual patients could not be personally identified, thus maintaining confidentiality. Data for research purposes were accessed on multiple occasions between November 16, 2022, and November 16, 2023, following the receipt of IRB approval.

## Results

The analysis included a total of 400 patient cycles with 202 cycles in aspiration pressures of 120 mmHg and 198 cycles in aspiration pressures of 150 mmHg. The baseline characteristics of the patients in the two groups are presented in Table 1. There was no statistically significant difference between the two groups in age, body mass index (BMI), serum anti-mullerian hormone (AMH), and antral follicle count (AFC). Type of infertility was comparable in both groups, and there were no statistically significant differences in the causes of infertility between the two groups.

**Table 1:** Baseline characteristics of patients.

	Aspiration pressure	Aspiration pressure	P value
	120 mmHg	150 mmHg	
Number of aspiration cycles	202	198	
Age (years)	38.6±4.6	38.5±4.4	0.774
BMI (kg/m^2^)	23.6±4.1	23.6±3.8	0.969
AMH (ng/ml)	2.0±2.7	2.4±2.9	0.115
Antral follicle count (AFC)	8.1±8.3	8.9±7.6	0.312
Type of infertility			0.512
Primary infertility	183 (90.6%)	183 (92.4%)	
Secondary infertility	19 (9.4%)	15 (7.6%)	
Cause of Infertility			
Male factor	47 (23.3%)	54 (27.3%)	0.357
Endometriosis	35 (17.3%)	37 (18.7%)	0.723
Ovulation disorder	26 (12.9%)	30 (15.2%)	0.511
Diminished ovarian reserve	97 (48.0%)	91 (46.0%)	0.680
Tubal factor	28 (13.9%)	35 (17.7%)	0.295
Uterine factor	48 (23.8%)	49 (24.7%)	0.818
Unexplained	14 (6.9%)	18 (9.1%)	0.426
Stimulation days	8.0±2.9	8.3±3.4	0.348
Total dose of gonadotropins (IU)	2842.9±1619.7	2647.2±1537.3	0.216
Number of follicle size ≥14mm	3.9±3.1	4.0±3.3	0.697
Type of fertilization			0.103
Conventional IVF (%)	79 (39.1%)	62(31.3%)	
ICSI (%)	123 (60.9%)	136 (68.7%)	

* continuous variables are presented as mean±SD, categorical variables as n (%)

Stimulation days (p = 0.348), total gonadotropin dose (p = 0.216), and number of follicles ≥14mm (p = 0.697) were not significantly different between aspiration pressure of 120 mmHg and 150 mmHg. There was no significant difference in type of fertilization between the two groups (p = 0.103) (Table 1). There were statistically significant differences between the two groups in the number of retrieved oocytes (6.3±5.2 vs. 7.7±6.7, p = 0.018) and mature oocytes (4.4±3.7 vs. 5.6±5.3, p = 0.011). The number of embryos yielded and good-grade embryos was significantly higher in the 150 mmHg group compared to the 120 mmHg group (3.3±3.1 vs. 4.2±3.9, p = 0.011 and 1.0±1.6 vs. 1.5±2.6, p = 0.031, respectively) (Table 2).

**Table 2:** Outcomes of controlled ovarian stimulation (COS).

	Aspiration pressure	Aspiration pressure	P-value
	120 mmHg	150 mmHg	
Number of retrieved oocytes	6.3±5.2	7.7±6.7	0.018
Number of fractured oocytes	0.2±1.0	0.2±0.6	0.889
Number of mature oocytes	4.4±3.7	5.6±5.3	0.011
Number of embryos	3.3±3.1	4.2±3.9	0.011
Number of good-grade embryos	1.0±1.6	1.5±2.6	0.031

* continuous variables are presented as mean±SD

The distribution of embryo stages at transfer did not differ significantly between the groups (p = 0.339). In the 120 mmHg group, 45/48 (93.7%) were cleavage-stage and 3/48 (6.3%) were blastocyst transfers, compared to 50/57 (87.7%) cleavage-stage and 7/57 (12.3%) blastocyst transfers in the 150 mmHg group. There was no significant difference in type of ET cycles between the two groups (p = 0.793). The percentage of fresh embryo transfer cycles was 60.4% in the 120 mmHg group and 57.9% in the 150 mmHg group, while frozen-thawed embryo transfer cycles accounted for 39.6% and 42.1% of the cycles, respectively. The number of embryos transferred was similar between the two groups, with a mean of 2.2±0.7 embryos in the 120 mmHg group and 2.0±0.7 embryos in the 150 mmHg group (p = 0.288). When analyzing the quality of embryos, the 120 mmHg group had 41.8% good-grade, 39.8% moderate-grade, and 18.4% poor-grade embryos, while the 150 mmHg groups showed 49.2% good-grade, 36.7% moderate-grade, and 14.1% poor-grade embryos. The distribution of cleavage and blastocyst and their grades are shown in S1 Table. The mean number of good-grade embryos transferred was comparable between the groups (0.9±0.8 vs. 1.0±0.9, p = 0.533).

There was no statistically significant difference in the clinical pregnancy rate between the two groups. The clinical pregnancy rate was 22.9% (11/48) in the 120 mmHg group and 24.6% (14/57) in the 150 mmHg group (p = 0.844). Similarly, there was no significant difference in the live birth rate, with rates of 10.4% (5/48) in the 120 mmHg group and 14.0% (8/57) in the 150 mmHg group (p = 0.575) (Table 3).

**Table 3:** Distribution and pregnancy outcomes in the first embryo transfer cycle.

	Aspiration pressure	Aspiration pressure	P value
	120 mmHg	150 mmHg	
Number of first ET cycles	48	57	
Number of cleavage-stage ET cycles	45 (93.7%)	50 (87.7%)	0.339^¥^
Number of blastocyst ET cycles	3 (6.3%)	7 (12.3%)	
Type of ET cycles			0.793
Fresh ET cycles	29 (60.4%)	33 (57.9%)	
Frozen-thawed ET cycles	19 (39.6%)	24 (42.1%)	
Grade of embryos	98	120	
Number of good-grade embryo	41 (41.8%)	59 (49.2%)	
Number of moderate-grade embryo	39 (39.8%)	44 (36.7%)	
Number of poor-grade embryo	18 (18.4%)	17 (14.1%)	
Number of embryos transferred	2.2±0.7	2.0±0.7	0.288
Number of good grade embryos transferred	0.9±0.8	1.0±0.9	0.533
Clinical pregnancy rate (%)	22.9% (11/48)	24.6% (14/57)	0.844
Live birth rate (%)	10.4% (5/48)	14.0% (8/57)	0.575

* continuous variables were presented as mean±SD, categorical variables as n (%)

^¥^Fisher’s exact test was performed

In the logistic regression analysis for clinical pregnancy and live birth rates, the odds ratios (OR) and adjusted odds ratios (aOR) were determined for each variable. For Age, serum AMH, AFC, and BMI, no statistically significant associations were observed with clinical pregnancy rate or live birth rate. The previous live birth showed no significant association with the clinical pregnancy rate; however, there was a statistically significant association with the live birth rate (p = 0.011). The factors contributing to live birth were identified as age, previous live birth and number of embryos transferred; the number of embryos transferred were also found to significantly impact clinical pregnancy after conducting multivariate logistic regression analysis. When comparing the grade of embryos transferred, good grades, and other grades, no significant associations were observed with clinical pregnancy rate or live birth rate (p = 0.520 and p = 0.361). The aspiration pressure of 120 mmHg compared to 150 mmHg showed no significant associations with clinical pregnancy rate or live birth rate (p = 0.519 and p = 0.170) (Table 4).

**Table 4:** Logistic regression analysis with odds ratios and adjusted odds ratios for clinical pregnancy rate and live birth rate.

	Clinical pregnancy rate				Live birth rate			
	OR (95% CI)	P-value	aOR (95% CI)	P-value	OR (95% CI)	P-value	aOR (95% CI)	P-value
Age (years)	0.959 (0.869–1.058)	0.401	0.886 (0.780–1.007)	0.065	0.959 (0.845–1.088)	0.512	0.794 (0.649–0.973)	0.026
BMI (kg/m^2^)	1.001 (0.893–1.123)	0.981	1.012 (0.891–1.100)	0.850	0.904 (0.765–1.068)	0.236	0.875 (0.705–1.085)	0.224
Serum AMH (ng/mL)	0.979 (0.774–1.238)	0.856	0.939 (0.626–1.408)	0.761	0.855 (0.599–1.220)	0.387	0.897 (0.474–1.699)	0.739
Antral follicle count	0.991 (0.924–1.062)	0.789	0.973 (0.861–1.100)	0.664	0.947 (0.849–1.055)	0.322	0.867 (0.701–1.074)	0.192
Previous live birth	1.714 (0.470–6.258)	0.415	3.561 (0.800–15.842)	0.095	2.767 (0.641–11.938)	0.173	13.734 (1.832–102.970)	0.011
Number of embryos transferred	2.070 (1.033–4.146)	0.040	2.957 (1.260–6.940)	0.013	2.805 (1.079–7.289)	0.034	7.290 (1.595–33.327)	0.010
Grade of embryos transferred								
Good grade	1.462 (0.546–3.915)	0.450	1.422 (0.486–4.164)	0.520	1.864 (0.479–7.255)	0.369	2.145 (0.418–11.011)	0.361
Other grade	1		1		1		1	
Aspiration pressure								
120 mmHg	0.913 (0.370–2.254)	0.844	0.725 (0.272–1.930)	0.519	0.712 (0.271–2.341)	0.576	0.370 (0.089–1.532)	0.170
150 mmHg	1		1		1		1	

## Discussion

This study compared the oocyte recovery, oocyte damage, embryo development, and live birth rate according to the aspiration pressure of the suction pump during oocyte retrieval in the IVF-ET cycle of infertile women. This study showed for the first time that the number of retrieved oocytes, mature oocytes, and embryos were significantly higher in aspiration pressure 150mmHg compared to 120mmHg without affecting oocyte damage and live birth rate. One study found that in infertile women with low ovarian reserve, the group that aspirated at 140 mmHg without flushing the follicles had significantly higher oocyte yield and pregnancy rate than the group that aspirated at 120 mmHg and then flushed the follicles [18]. However, it is difficult to generalize the study results to infertile patients with normal ovarian reserve because the study was conducted on patients with low ovarian reserve. Since the group that performed follicular flushing was compared with the group that did not, there is a critical problem in that it is difficult to determine whether the difference in results is due to the aspiration pressure. There is no information provided on the woman’s age, number of embryos transferred, and the grade of embryo, which directly affect the pregnancy rate. The sample size was small, and confounding variables such as age, ovarian reserve, cause of infertility, and dose of gonadotropin that affect the number of oocytes retrieved and embryos obtained were not properly controlled, making the results unreliable. It does not describe the exclusion criteria for study subjects, the pump used, the number of patients who underwent oocyte retrieval but were unable to aspirate the oocytes, the inner diameter and length of the aspiration needle, whether it was a single lumen. Even if the aspiration pressure is the same, the larger the inner diameter and shorter the length of the aspiration needle, the higher the velocity of fluid within the needle and the higher the rate of oocyte damage [8]. In addition, this study compared the clinical pregnancy rate between the two groups and did not compare the live birth rate.

In general, the higher the aspiration pressure, the higher the oocyte recovery rate, but the number of oocytes surrounded by compact cumulus decreases while the proportion of damaged oocytes increases [19]. The results of this study showed that the number of retrieved and mature oocytes was higher in the group aspirated with a pressure of 150 mmHg, and subsequently the number of embryos produced was also higher. Theoretically, as only 5% of the total aspiration pressure is applied to the tip of the needle located in the follicular fluid, the absolute difference in pressure in the follicular fluid between the two groups is only 1.5 mmHg [8]. Nevertheless, this small difference in pressure caused a difference in the average number of oocytes retrieved by 1.4 and the number of embryos produced by 0.9. This has important clinical significance because the cumulative live birth rate (CLBR) continues to increase as the number of oocytes retrieved and the number of embryos produced increases [20–22]. In this study, only the live birth rate in the first cycle was compared because the patients included in the analysis did not use all of the remaining embryos, but if CLBR was compared, it may have been significantly higher in the 150 mmHg group.

In terms of oocyte damage, there was no difference in the fracture rate of the zona pellucida between the two groups. First, this is believed to be because the study’s aspiration pressure comparison was in the 100 mmHg range, which is not a high pressure level. Although it was conducted for IVM by collecting immature oocytes, the rate of COC damage was higher, and the rate of transferable embryos and ongoing pregnancy rate were significantly lower when retrieved with an extremely high aspiration pressure of 300 mmHg compared to 180 mmHg [23]. Second, it is thought that the patients who participated in this study were mainly normal responders, and the quality and durability of COC were relatively good. It is known that the better the quality and durability of the COC, the better it can withstand high aspiration pressure without being damaged [8]. If this study had targeted poor responders with poor COC quality, there may have been differences in the rate of zona fracture. Third, as long as the COC remains intact, the zona pellucida, the innermost structure in contact with the oocyte, is likely to withstand relatively high aspiration pressure, even with a slight increase in pressure. Animal studies have shown that the proportion of oocytes damaged in the order of the outer cumulus cell, corona radiata, and zona pellucida increases as the aspiration pressure increases [8]. If the degree of denudation of corona radiata or cumulus cells located further outside the zona pellucida had been analyzed in detail, there may have been differences between the two groups.

As the aspiration pressure goes up, the size of the turbulent flow grows when the follicular fluid enters the suction needle. Also, the difference in velocity of the laminar flow grows even after it enters the needle, which causes the cumulus and corona radiata to break apart due to shear stress force [9]. In this study, a 17-gauge (inner diameter of 1mm) needle was used. Human COC occupies approximately one fourth of the inner diameter of the needle, inevitably exposing its outer surface to different shear stress forces. Consequently, the outer layer of COC is pulled, and the COC rotates and advances by hitting the inner wall of the needle, leading to the stripping of cumulus and, in severe cases, the fractures of zona pellucida [9, 15]. Because this was a retrospective study with no data on the morphology of the oocyte cumulus mass, it could not directly prove whether the rate of cumulus denudation and corona radiata damage was higher in the 150 mmHg group. However, the study indirectly demonstrated that there was no difference in oocyte competence, as evidenced by the similarity in clinical outcomes such as the number of high-grade embryos and the live birth rate between the two groups.

Firstly, the strengths of this study are that, although it is a retrospective design, the allocation to the two groups was random, so there is a low possibility of confounding bias, and since the patients do not know their own aspiration pressure, a blinding effect is applied, so there is little possibility for information bias. Second, patients who underwent flushing were excluded from the study, and analysis was performed only on patients who performed aspiration with a single lumen 17-gauge needle of the same length and inner diameter. Third, compared to previous studies, the sample size was large, clinically important live birth rates were compared, and all oocyte retrieval was performed by well-trained specialists. The limitation is that there was no information on the morphology of the oocyte cumulus mass, so it was not possible to directly prove whether the rate of cumulus denudation and corona radiata damage was higher in the 150mmHg group.

This study found that the two groups’ pregnancy outcomes, including the live birth rate, were comparable, necessitating further research to explore potential differences even at higher aspiration pressures in the future. Higher aspiration pressures theoretically allow for the aspiration of a larger volume of follicular fluid per time. Therefore, retrieving oocytes at a higher pressure can shorten the operating time and reduce the anesthetic dose if future research demonstrates that there is no difference in pregnancy results even with high aspiration pressure [18]. Although there was no difference in live birth rate between the two groups, research is needed to determine whether there is a difference in CLBR depending on aspiration pressure, as there was a significant difference in the number of retrieved oocytes, mature oocytes, and embryos produced. Additional research on aspiration pressure for other patient groups and clinical situations, such as oocyte retrieval from low-responders or elderly patients, use of a 19-gauge needle or double lumen needle, or aspiration of immature oocytes during IVM, should continue, and well-designed, large-scale RCTs are also needed. It is expected that research results related to the aspiration pressure of the pump will continue to be published and a standardized aspiration protocol will be established when retrieving oocytes using a pump.

## Supporting information

S1 Table Quality of embryos according to aspiration pressure. (DOCX)

S1 Dataset Raw data comparing 120 mmHg and 150 mmHg aspiration pressure for oocyte retrieval outcomes in IVF cycles. (XLSX)
