# Supplementary material for: Optimal aspiration pressure of suction pump for oocyte retrieval in infertile patients undergoing in vitro fertilization
Source: PLoS One. 2025 Jan 27;20(1):e0317812. doi: 10.1371/journal.pone.0317812 (PMC11771860; doi:10.1371/journal.pone.0317812)
Supplement: S1 Table — (DOCX) [file pone.0317812.s001.docx]

**S1 Table. Quality of embryos according to aspiration pressure**

|  | Aspiration pressure  120 mmHg | Aspiration pressure  150 mmHg |
| --- | --- | --- |
| Grade of cleavages  Number of good-grade cleavage  Number of moderate-grade cleavage  Number of poor-grade cleavage | 93  36  39  18 | 112  51  44  17 |
| Grade of blastocysts  Number of good-grade blastocyst  Number of moderate-grade blastocyst | 5  5  0 | 8  8  0 |
| Number of poor-grade blastocyst | 0 | 0 |
